# New technologies in airway management: A review

**DOI:** 10.1097/MD.0000000000032084

**Published:** 2022-12-02

**Authors:** Ana Vasconcelos Pereira, André Vicente Simões, Luísa Rego, João Gonçalves Pereira

**Affiliations:** a Anesthesiology Department, Hospital de Vila Franca de Xira, Vila Franca DE Xira, Portugal; b Intensive Care Department, Hospital de Vila Franca de Xira, Vila Franca DE Xira, Portugal.

**Keywords:** artificial intelligence, assisted devices, difficult airway, intubation, videolaringoscopy

## Abstract

The evolution of medical knowledge and technological growth have contributed to the development of different techniques and devices for airway management. These appear to play a role in optimizing the number of attempts and overall success, ultimately reducing the negative consequences of airway manipulation. In this literature review, we highlight the recent evidence regarding new technologies applied to airway management.

Before intubation, every patient should have an individualized structured airway management plan. Technology can help with both airway evaluation and tracheal intubation. Point-of-care cervical ultrasound and artificial intelligence models with automated facial analysis have been used to predict difficult airways. Various devices can be used in airway management. This includes a robotic video endoscope that guides intubation based on real image recognition, a laryngeal mask with a non-inflatable cuff that tries to reduce local complications, video laryngeal masks that are able to confirm the correct position and facilitate intubation, Viescope™, a videolaryngoscope developed for combat medicine with a unique circular blade, a system that uses cervical transillumination for glottis identification in difficult airways and Vivasight SL™ tracheal tube, which has a high-resolution camera at its tip guaranteeing visual assurance of tube position as well as guiding bronchial blocker position.

To conclude, we detailed the challenges in airway management outside the operating room as well as described suction-assisted laryngoscopy and airway decontamination technique for contaminated airways.

Further research in the clinical setting is recommended to better support the use of these technologies

## 1. Introduction

The 4^th^ National Audit Project (NAP4) of the Royal College of Anaesthetists and Difficult Airway Society is the world’s largest audit of airway complications. NAP4 prospectively examined a large cohort of major airway complications that lead to death, brain damage, emergency surgical airway or unexpected intensive care unit admission in different settings for 1 year, across the United Kingdom. Major complications of airway management occurred at a rate of 46.3 (95% CI 38.4–54.2) per million cases during general anesthesia. These incidences were both more severe and common in the emergency and intensive care departments.^[1,2]^

To reduce the principal adverse outcomes associated with difficult airways, practice guidelines were published with recommendations intended to be used by any individual who performs airway management.^[3]^ The evolution of medical knowledge and technology contributed to the revision of these guidelines.^[4]^

The adoption of novel technologies in daily practice requires detailed investigation to ensure that their use produces intended patient-centered and measurable outcomes without negative consequences.^[5]^

This literature review includes published evidence related to new technologies in airway management mainly developed in the last 5 years. Several questions have been explored, including: Is there a place for artificial intelligence in airway management? Can a trainee be teleguided during intubation? Is there a difference between tracheal intubation and insertion of supraglottic devices in emergency airway management? Can a video laryngeal mask be more effective? How can ultrasonography be useful for airway evaluation?

## 2. Methods

The literature search was divided into 2 separate occasions: December 27, 2021, and October 18, 2022, using recognized electronic databases: PubMed, Cochrane Database of Systematic Reviews and Tripdatabase. The following keywords were used in the literature search: airway, airway management, intubation, supraglottic devices, technology, technology update, new technologies, artificial intelligence and teleguidance. The search was limited to guidelines, review articles, observational studies, controlled trials, meta-analyses, and systematic reviews, published between January 2017 and October 2022. Based on the initial list of articles selected from the referred databases, a manual search was performed to identify other articles of interest that were not captured by the initial electronic database search, such as those with historic value.

## 3. Discussion/observations

### 3.1. Point-of-care ultrasound in airway management

Airway management through endotracheal intubation is commonly performed in elective surgical procedures, intensive care, and in the emergency department.^[6]^ Point-of-care ultrasound (POCUS) imaging is an expanding area that has been recently applied to airway management, contributing to a reduction in morbimortality related to airway interventions. POCUS allows dynamic, quick, reliable, and real-time assessment of the airway. Furthermore, it is easy to learn with minimal training required.^[7]^

Traditional clinical examinations have limited predictive value in assessing difficult airways.^[8]^ POCUS allows rapid screening for difficult direct laryngoscopy, assesses the aspiration risk and identifies the cricothyroid membrane in patients with predicted difficulty that may need front-of-neck access.^[7]^

Measuring the distance from the skin to the epiglottis (DSE) is one of the most studied index tests to predict difficult direct laryngoscopy, with a sensitivity of 0.82 (95% CI, 0.74–0.87), specificity of 0.79 (0.70–0.87) and AUC of 0.87 (0.84–0.90). Patients with higher Cormack–Lehane grades had higher DSE. Despite the heterogeneity of cut off values between studies,^[9–11]^ a DSE > 2 to 2.5 cm seemed to identify difficult direct laryngoscopies.^[10]^

Other ultrasonography indicators for predicting difficult intubation were evaluated in a 2021 systematic review. Distance from skin to the epiglottis, distance from skin to the hyoid bone and hyomental distance were found to be correlated with difficult laryngoscopy.^[12]^ An anterior neck soft tissue measurement higher than 2.8 cm at the thyrohyoid membrane level is associated with difficult laryngoscopies (defined cut off value).^[13]^

POCUS can also be used to confirm endotracheal tube position, especially in critically ill patients. According to the 2021 European Resuscitation Council Guidelines, continuous-waveform capnography is the most sensitive and specific method for confirming and continuously monitoring the position of an endotracheal tube during cardiac arrest. Airway ultrasound performed by skilled operators is also referred to as a method for confirming tracheal tube position.^[14]^

The 2015 American Heart Association Guidelines Update for Cardiopulmonary Resuscitation and Emergency Cardiovascular Care also made a class II recommendation regarding the use of ultrasound to identify endotracheal or esophageal intubation when continuous waveform capnometry is unavailable or unreliable.^[15]^

The assessment consists in placing the linear ultrasound probe transversely across the suprasternal notch and identifying the endotracheal tube cuff balloon. If there is esophageal intubation there will be a “double tract sign” with 2 air-filled structures with acoustic shadowing. This evaluation can be further supported by thoracic ultrasonography, which confirms bilateral lung sliding.^[7]^ The average time to confirmation is 13 seconds, with a demonstrated sensitivity of 0.99 (95% CI, 0.98–0.99) and specificity of 0.97 (95% CI, 0.92–0.99).^[6]^

### 3.2. Artificial intelligence in airway management

Airway management in the emergency department is often challenging because of the physiologically difficult airways caused by several factors such as hemodynamic instability, vomiting, facial/cervical trauma and cervical immobilization. Therefore, it is essential to achieve rapid and successful intubation in high-risk patients.^[16]^

Endotracheal intubation may be a life-saving procedure that requires technical skills. Many rural and remote areas, lack experienced airway providers.^[17]^ In this setting, the use of artificial intelligence (AI) could be useful for airway management.

Dhananchey et al proposed a conceptual model for an intubation device based on clinical technology-research integration. The model started with new inputs (data from patients) that were reprocessed with AI, resulting in outputs such as the classification/clustering of patients and visual decision guidance^.[18]^

Robotic endoscope-automated laryngeal imaging for tracheal intubation (REALITI) is a video-endoscopic stylet that guides endotracheal intubation. The bending motion of the endoscope tip can be controlled manually or bent automatically towards the glottis owing to real image recognition. This prototype was first used in a simulated environment in 2020, comparing the device’s ability to intubate a manikin by experienced anesthesiologists and participants without medical training.^[19]^ The prototype, operating in automated mode, records a glottic image that resembles anatomic airway images stored in the electronic database, then steers the endoscope tip into the trachea, allowing successful tracheal intubation.^[11]^ In the automated mode, after six attempts each, the anesthesiologists and non-trained personnel succeeded with 95% and 100% automated insertions, respectively. The duration of insertion was also similar between groups.^[19]^

Hayasaka et al also published an AI model that could be applied to tracheal intubation performed by inexperienced medical staff. Sixteen facial images from different patients were obtained and classified based on expected difficulty. Subsequently, an AI classification model was created based on deep learning (convolutional neural network). The AI model recognized facial contours and then identified expected intubation difficulties, with 80.5% accuracy, 81.8% sensitivity, 83.3% specificity and an AUC of 0.864 (CI 95%, 0.731–0.969).^[20]^

Other studies have approached the role of machine learning as a complement to the physical exam, performing automatic facial analysis and detecting morphological traits related to difficult airways.^[21,22]^ It has also been applied to monitoring pediatric airways, enhancing the detection of critical incidents and providing early warnings to the clinician.^[22]^

### 3.3. Teleguided technology for intubation

One of the first clinical cases of teleguidance in medicine was published 48 years ago.^[23]^ This technology was reexplored during the coronavirus disease pandemic as a way to minimize provider exposure.^[17]^

A scoping review of teleguided technology for endotracheal intubation elucidated the feasibility, barriers, and complications inherent to its use. Teleguided facilitated intubation appears to be as effective as in-person supervision, with no further complications. It also has educational purposes, allowing progressive autonomy for the trainees.^[17]^

A randomized clinical simulation trial assessed the application of tele-glass technology to assist endotracheal intubation. Fifteen nurses from the emergency department were randomized to a group that simulated intubation with telemedicine assistance or a control group (without assistance). The telemedicine group had an intubation success rate of 96%, whilst the control group only achieved 72% (*P* = .024), also with significantly less time required to intubate [mean difference 94.3 seconds (IC 95%, 40.69–147.96 seconds), *P* = .001].^[24]^

In one case report, tele-glasses were used in the perioperative setting, for airway assessment. During anesthesia induction, tele-glasses also recorded laryngoscopy view, which was attached to an individual record, for both teaching and self-assessment purposes.^[25,26]^

### 3.4. The future of extraglottic devices

The use of supraglottic airway devices (SADs) has increased exponentially since its introduction in 1982.^[27]^ According to the NAP4, airway management was carried out using a supraglottic airway in 56% of general anesthetics administered every year (in the United Kingdom National Health Service).^[2]^ The use of SADs for rescue airway management is also widely recommended in several difficult airway guidelines.^[4,28,29]^ Observational studies indicated a 65% to 100% success rate related to SADs insertion and intubation in difficult airways.^[4]^ They not only permit oxygenation (when the initial intubation attempt fails) but also serve as a conduit for tracheal intubation.^[27]^

Second-generation SADs incorporated separate ventilation and gastric access tubes. The definition of a third generation SADs is not consensual, usually referred to as a mask with a self-energizing sealing cuff (Baska Mask™) or as vision-guided SADs.^[30,31]^

The Baska Mask™ is an extraglottic device that allows a peri-laryngeal seal with a self-energizing sealing cuff. A non-inflatable cuff reduces the risk of oropharyngeal tissue and nerve damage, related to cuff overinflation. It has 2 gastric drain tubes, providing better protection against gastric content aspiration.^[30,32]^ Although Baska Mask™ is more challenging to insert, it achieves higher mean oropharyngeal seal pressure when compared to Supreme laryngeal mask™ (33.28 ± 6.80 cm H_2_O vs 27.47 ± 2.34 cm H_2_O, *P* < .001).^[33]^

The intubating laryngeal tube (ILTS-D™) is a 2^nd^ generation extraglottic device that allows laryngeal tube suction and the possibility of secondary tracheal intubation. The revised model, ITLS-D2™ was tested against the laryngeal mask Fastrach™ in a randomized simulation study. The insertion success rate was 100% with a median insertion time of 13s (IQR: 12–15 seconds, *P* = .592).^[34]^ In another 2022 prospective multicenter randomized non-inferiority study, ILTS-D™ was again compared to Fastrach™, showing a lower overall intubation success rate (91.8% vs 70.0%, *P* = .006).^[35]^

In most cases, SADs are placed sub-optimally according to the theory of correct placement, device size and insertion depth.^[31,36]^ Clinical assessment may not ascertain correct positioning after blind insertion. This motivated the development of video laryngeal masks, which combine the features of a 2^nd^ generation SAD along with the ability to place and intubate under direct vision. Moreover, during intubation, these devices guarantee continuous oxygenation and allow visual inspection of the supraglottic area, distal to the cuff.^[36,37]^

The Video Laryngeal Mask™ and SafeLM™ are 2 examples of video laryngeal masks available. Their structure includes a disposable 2nd generation SAD with a silicone cuff, anatomically curved tube, reusable videoscope and a monitoring screen.^[36]^

The main advantages of these new airway devices are easy insertion under direct vision, reduced airway damage, capacity to pass an endotracheal tube through the SAD, recording capacity, and ability to dissociate the SAD from the videoscope once placed, allowing reuse of the videoscope.^[36]^

When compared to igel™ combined with flexible bronchoscopy guided intubation, a video laryngeal mask SaCoVLM™ provided a direct vision of the laryngeal inlet, faster intubation and fewer post-intubation complications.^[37]^ Clinical studies are necessary to fully elucidate the advantages and possible limitations of this strategy.

### 3.5. Videolaryngoscopes

Laryngoscopy is a key component of airway management in different settings such as critical care, anesthesia, and emergency medicine. Laryngoscopy technology has greatly expanded in the last decade.^[38]^ The ideal intubation scope should be safe, efficient, portable, reliable and cost-effective. Videolaryngoscopes (VL) provide an indirect oropharyngeal view using a light-emitting source and a sensor that converts light into electrical signals.^[38]^

According to the Difficult Airway Society 2015 guidelines, all anesthetists should be skilled in the use of a VL.^[29]^ Videolaryngoscopy is associated with improved laryngeal views, a higher intubation success rate and a higher success at the first attempt.^[4,38]^ A 2016 systematic review showed fewer failed intubations (OR 0.35, 95% CI 0.19–0.65), especially among difficult airways (OR 0.28, 95% CI 0.15–0.55).^[39]^ However, it remains controversial whether VL should be employed as a first-line method for tracheal intubation instead of direct laryngoscopy.^[38]^

Vie Scope™ is a new video scope for airway management that was originally designed for combat medicine owing to its ready-to-use quality. Vie Scope™ features a closed circular tube with a beveled end, resembling a Miller-shaped laryngoscope blade, that is transparent and illuminated. It allows direct glottis visualization and facilitates endotracheal intubation through a bougie.^[40,41]^

Vie Scope™ was compared with GlideScope™ and Macintosh™ laryngoscopies in a randomized controlled simulation trial. Thirty-five anesthesiologists performed endotracheal intubation using a difficult airway model. Vie Scope™ showed better glottis visualization than direct laryngoscopy. However, the mandatory use of a bougie in Vie Scope™ jeopardized its potential benefits. The rate of correct tube positioning was similar between devices, but both GlideScope™ and Macintosh™ laryngoscopy had a shorter time until intubation and ventilation, compared to Vie Scope™ (*P* < .001 for both).^[41]^ Other randomized single-blinded cross-over simulation trial evaluated the intubation performance of direct laryngoscopy and Vie Scope™. Forty-two paramedics tested the devices in a simulated difficult airway scenario. Vie Scope™ again, offered a better glottic view, but this time also allowed for higher success in the first attempt and shorter intubation time.^[42]^ Vie Scope™ also enabled adequate endotracheal intubation in a simulated pediatric setting.^[43]^ Further clinical studies are needed to evaluate the role of this device in clinical practice.

The infrared red intubation system (IRRIS) was first described in 2017 by Kristensen et al^[44]^ The technique consists of a small infrared light source (wavelength between 730 nm and 1000 nm), placed on the anterior cervical surface, over the cricothyroid membrane. The device emits infrared red light through the skin of the patient skin to the subglottic space. Then, a videolaryngoscope (whose structure does not filter that wavelength) is placed in the airway. The videoscope will display a bright light emerging from the glottis, guiding the path. IRRIS requires the use of indirect laryngoscopy, considering the type of radiation emitted.^[44,45]^ A single-center, prospective trial assessed the efficacy of IRRIS for laryngeal identification in obese patients proposed for bariatric surgery. The median larynx recognition time was independent of the weight. Tracheal intubation through IRRIS lasted 50 seconds (IQR 20–100), with the lowest oximetry of 98%.^[46]^ IRRIS technique has also been used in combination with flexible bronchoscopy.^[47]^

### 3.6. Endotracheal tubes and conductors

Endotracheal tubes are cylindrical structures made of polyvinylchloride that are placed through the glottis into the trachea, providing oxygen and inhaled anesthetics, and securing a definitive airway. The tracheal tube structure has been modified to better adapt to different clinical settings and surgical procedures.^[48]^

The VivaSight™ SL is a single-lumen endotracheal tube with an integrated high-resolution camera at its tip. The integrated camera provides visual assurance during intubation, enables continuous intraoperative tube evaluation, and monitors the bronchial blockers placement. The device is available in sizes of 7.0 to 8.0 mm (internal diameter).^[49]^

A randomized crossover simulation trial compared intubation performed using the VivaSight™ SL and conventional laryngoscopy. Sixty-seven novice paramedics attempted oral intubation using both techniques in 3 different scenarios, with progressive airway complexity. In the third scenario, with a trauma victim in need of cervical stabilization, the overall success was similar, but the time to intubation, success at the first attempt and glottis visualization were significantly better with VivaSight™.^[50]^

A meta-analysis of simulation studies, with participants with no previous intubation experience, compared VivaSight™ SL to conventional “blind” intubation. VivaSight™ performed better, with a higher success rate, shorter insertion time and better laryngeal view, in difficult airways.^[51]^ Its use during cardiopulmonary resuscitation was tested in a simulation study. Its effectiveness at the first attempt was 100%, with a median time to intubate of 25.5 seconds (IQR; 24–28.5 seconds).^[52]^ A randomized, crossover cadaver trial, showed, once more, its good performance during continuous chest compressions.^[53]^

The VivaSight™ SL has also been tested in clinical trials. A prospective study of 27 morbidly obese patients undergoing laparoscopic sleeve gastrectomy compared the clinical performance of VivaSight™ SL to conventional endotracheal intubation. The success on the first attempt was similar; the time to intubation was longer with VivaSight™ SL, with a mean time of 29 ± 10 seconds (*P* = .02); however, local complications were fewer.^[54]^

According to the Difficult Airway Society 2015 guidelines, gum elastic bougies are devices used to facilitate tracheal intubation, in cases of grade 2 or 3a Cormack-Lehane classification. Blind bougie insertion (Cormack–Lehane classification 3b or 4) is not recommended, as it is associated with laryngeal trauma.^[29]^ The use of a laryngoscope while the bougie is in the trachea, facilitates intubation.^[55]^ Bougies are used to guide the placement of supraglottic devices, as well as a “railroad” for tracheal tubes in scalpel cricothyroidotomy.

The use of a bougie device in difficult airway scenarios has variable success rates. Most reported difficulties are the inability to pass the hypopharynx (52%) or the endotracheal tube over the bougie (24%).^[29,56]^ A new flexible tip bougie was designed to overcome those difficulties. It has an integrated slider that moves its tip along the surface. The insertion technique is the same as that used for the standard bougie. An observational, cross-over simulation trial compared the flexible tip bougie with the standard bougie in 6 different airway scenarios. In more difficult airways, the flexible tip bougie achieved a similar success rate, but with fewer intubation attempts and less local complications.^[57]^

### 3.7. Emergency airway management outside of the operating room

Emergency airway management outside of the operating room is challenging. Along with anatomically difficult airways, physiological and situational challenges are common. Minimal physiological reserves pose a higher risk of morbimortality.^[58]^

Limited space, inadequate lightning, difficult airway assessment/poor cooperation, full stomach, facial and cervical trauma, poor physiological reserve, limited training and inexperienced providers are some of the challenges. Several recommendations should be considered when managing emergency airways, such as adequate hemodynamic monitoring, continuous capnometry, availability of functioning high-efficiency suction devices, and the implementation of checklists.

Emesis may occur in up to one third of cardiac arrests, and its presence decreases the odds of survival. Any approach that acts on the prevention of emesis improves the chances of survival following out-of-hospital cardiac arrest.^[59]^ Bleeding in the upper airway is another important cause of airway-related death.^[2]^

Suction-assisted laryngoscopy and airway decontamination, known as the SALAD technique, advocates the use of suction along with emergency airway management to address the problem of massive airway contamination. The SALAD technique may be considered in a clinical setting such as regurgitation of gastric contents, copious secretions, post-operative upper airway bleeding and upper gastrointestinal hemorrhage.^[60]^ It has also been used outside the operating room, in cases of upper airway hemorrhage or trauma.^[5]^

The rigid suction catheter is inserted into the mouth and swept side to side; then it is used to displace the structures of the upper airway (tongue elevation) until the blade of the laryngoscope has been optimally positioned. During intubation, the catheter maintains continuous suction in the hypopharynx.^[5,60]^ A bougie can be used to aid in intubation.

The effectiveness of the SALAD technique performed by paramedics using a soiled airway manikin model was evaluated in the SALTIATED trial. More paramedics were able to intubate on their first attempt with the SALAD technique (90.2% vs 53.7%, 95% CI 24–49.1%, *P* < .001).^[61]^ Structured educational interventions improved outcomes from this technique (time to intubation and number of intubation attempts).^[60,62]^ Compromised oxygenation during intubation and suboptimal visualization of the larynx, due to the suction catheter, with inadvertent esophageal intubation are some downfalls of the SALAD technique. An technically proficient operator is fundamental.^[60]^

Optimal cardiopulmonary resuscitation is associated with better outcomes. Therefore, an effective early airway management is essential. Traditional teaching suggests that tracheal intubation is the most effective way to manage the airway during out-of-hospital advanced cardiovascular life support. But what if a supraglottic device is as much effective? A multicenter, randomized controlled trial aimed to determine whether the supraglottic airway of the igel™ is superior to tracheal intubation in non-traumatic out-of-hospital cardiac arrest. There were no significant differences in complications, costs, outcomes or overall cost-effectiveness between groups.^[63]^

## 4. Conclusions

The implementation of new devices, techniques and technologies in airway management should consider population, environment, and locally available resources. Regardless of scientific discoveries and technologies, an airway management plan should be well-structured. Further research in the clinical setting is fundamental to support the use of these new technologies in the operating room, intensive care, and emergency department.

## Author contributions

**Conceptualization:** Ana Vasconcelos Pereira, André Vicente Simões, João Gonçalves Pereira.

**Data curation:** Ana Vasconcelos Pereira.

**Formal analysis:** Ana Vasconcelos Pereira.

**Investigation:** Ana Vasconcelos Pereira.

**Methodology:** Ana Vasconcelos Pereira.

**Project administration:** Ana Vasconcelos Pereira.

**Resources:** Ana Vasconcelos Pereira.

**Software:** Ana Vasconcelos Pereira.

**Supervision:** André Vicente Simões, João Gonçalves Pereira.

**Validation:** André Vicente Simões, Luísa Rego, João Gonçalves Pereira.

**Visualization:** Luísa Rego.

**Writing – original draft:** Ana Vasconcelos Pereira.

**Writing – review & editing:** André Vicente Simões, Luísa Rego, João Gonçalves Pereira.
